# Biodegradation of 2-hydroxyl-1,4 naphthoquinone (lawsone) by Pseudomonas taiwanensis LH-3 isolated from activated sludge

**DOI:** 10.1038/s41598-017-06338-1

**Published:** 2017-07-28

**Authors:** Li Yang, Tianming Cai, Dahu Ding, Tianjin Cai, Canlan Jiang, Hua Li, Qian Yang, Liwei Chen

**Affiliations:** 1 College of Resources and Environmental Sciences, Nanjing Agricultural University, Nanjing, 210095 China; 2 College of Geographic and Biologic Information Nanjing University of Posts and Telecommunications, Nanjing, 210023 China

## Abstract

2-hydroxy-1,4 naphthoquinone (lawsone) is widely used and induces environmental pollutions during its production and application. In the present study, a lawsone-degrading bacterium strain, LH-3 was successfully isolated from the activated sludge. Based on the 16S rRNA gene analysis, the strain LH-3 phylogenetically belonged to the *Pseudomonas taiwanensis*. It could degrade 200 mg L^−1^ lawsone completely in 9 h with an inoculum quantity of 1% (v/v). The effects of environmental conditions on the degradation process and the degradation pathway were systematically investigated. LH-3 could maintain its high degradation efficiency under high salt condition. The identified intermediates of salicylic acid, 2-hydroxy-4-oxo-chroman-2-carboxylic acid, and catechol elucidated the potential degradation pathway. Furthermore, the immobilized LH-3 strain cells prepared with alginate gel and biochar performed excellent stability in nine successive degradation runs. It could sucessfully survive in laboratory scale sequencing batch reactor and become to be the dominant species. This study clearly revealed that LH-3 could serve as an attractive candidate for the microbial remediation of lawsone-containing wastewater.

## Introduction

2-hydroxy-1,4 naphthoquinone (lawsone) is a well-known compound occurred in the henna plant leaves. It could react chemically with the protein keratin in skin and hair via Michael addition, leading to a strong permanent stain^1^. For thousands of years, it was a popular dye used for dyeing hair, skin and nails^2^. Recently, its bactericidal, fungicidal, antimalarial and cytostatic properties were reported^3–5^. More importantly, its role as a redox mediator was elucidated in many works^6, 7^. As a result, its application field was greatly extended to the pharmaceutical, chemical and other industries^8–11^. Inevitably, the lawsone entered the surrounding environments along with the wastewater and might pose potential threats to aquatic organisms^12, 13^. According to the summary report of the National Center for Biotechnology Information (NCBI), the lawsone could cause skin irritation and serious eye irritation. Wright *et al*.^14^ indicated that a broad spectrum of free-living phytoplankton would be controlled by the low concentrations of naphthoquinones (as low as mg/L). Sauriasari *et al*.^15^ investigated the cytotoxicity of lawsone and pointed out that it was not mutagenic, but toxic to the cells in dose-dependent manner. Though the environmental fate (transformation, degradation) of the lawsone is largely unknown up until now, the high concentration of lawsone in some industrial wastewater still called for effective remediation.

It is well believed that biodegradation method with the assistant of intrinsic microorganisms is an economical and eco-friendly method for the remediation of contaminated environmental media^16–19^. Through isolation and enrichment, the specific pollutant-degrading microorganism could be obtained from various media including soil and sludge^16, 20^. For example, Schmidt S.K.^21^ successfully isolated a strain *Pseudomonas* J1 from soil and found it could degrade lawsone rapidly. Similarly, Wessendorf *et al*.^22^ reported the degradation of lawsone by *Pseudomonas putida* L2 isolated from humus and proposed a degradation pathway. Muller and Lingens^23^ reported that the *Pseudomonas putida* J1 and J2 enriched from soil could degrade 1,4-naphthoquinone, lawsone, and 2-chloro-1,4-naphthoquinone. However, to the best of our knowledge, the publication related to the isolation and characterization of lawsone-degrading strain from activated sludge was very scarce up to now.

The aims of this study were mainly (i) to isolate a bacteria from activated sludge that could effectively degrade lawsone; (ii) to investigate the biodegradation characteristics under different environmental conditions; (iii) to prepare the immobilized cells with alginate gel, and finally (iv) to elucidate the potential degradation pathway.

## Results and Discussion

### Isolation and Characterization of LH-3

**Figure 1:**
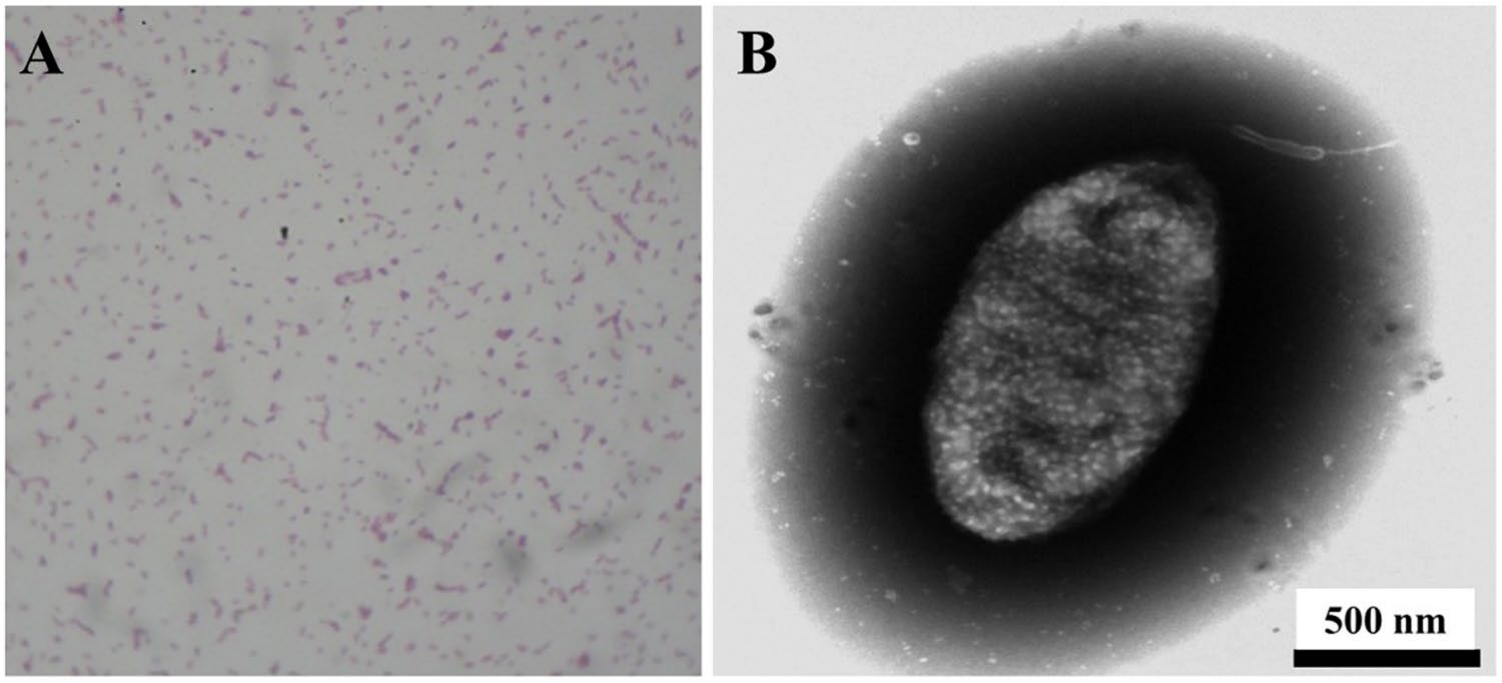
(**A**) The image of crystal-violet staining assay of LH-3 and (**B**) the TEM image of a single LH-3 cell.

**Figure 2:**
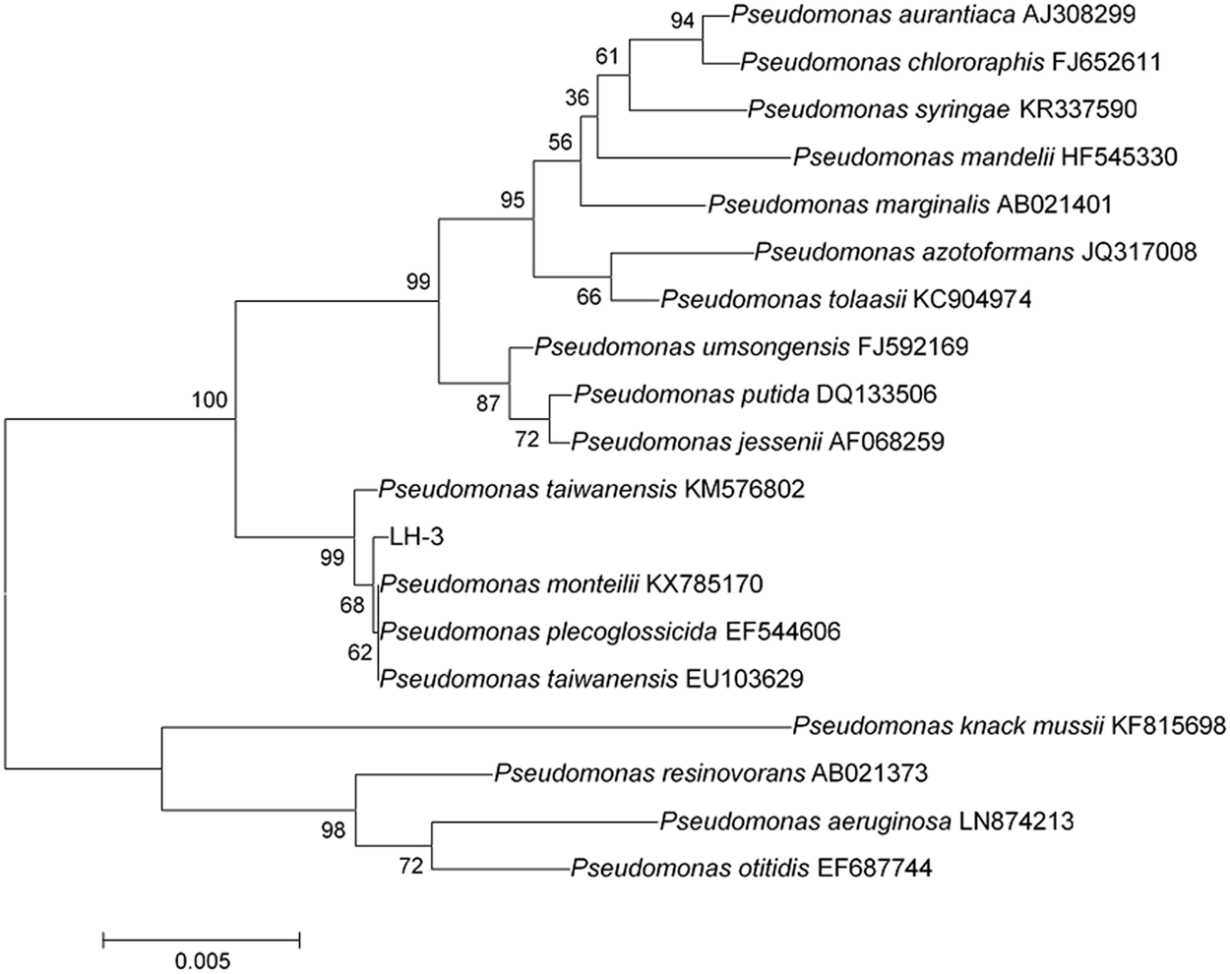
Phylogenetic tree based on the 16S rRNA gene sequences of strain LH-3 and the related species. The GenBank accession number for each microorganism used in the analysis was also given in the brackets. Bootstrap value obtained with 1000 samplings was indicated as percentage for the each branch. The scale bar indicated 0.005 substitutions per nucleotide position.

As mentioned, a pure colony, designated as LH-3, was successfully isolated from the activated sludge. The strain could grow using lawsone as the sole carbon source. As shown in Fig. S1, the colonies were small round and white-mucoid. Noting that, the area with colonies distributed clearly changed to colourless from orange, indicating the degradation of lawsone by the colonies. The results of crystal-violet staining assay further indicated that the strain LH-3 was a gram-negative bacterium (Fig. 1A). As depicted in Fig. 1B, the strain LH-3 was short-bar shaped and the size was around 1.5~2 µm. The 16S rDNA gene sequence results indicated that strain LH-3 formed a distinct lineage within the genus *Pseudomonas* (Fig. 2), showed 99.93% similarity to *Pseudomonas taiwanensis* BCRC17751(T). It is previously reported that the *Pseudomonas* species can degrade various organic compounds including dyes^24, 25^.

### Degradation Assay

**Figure 3:**
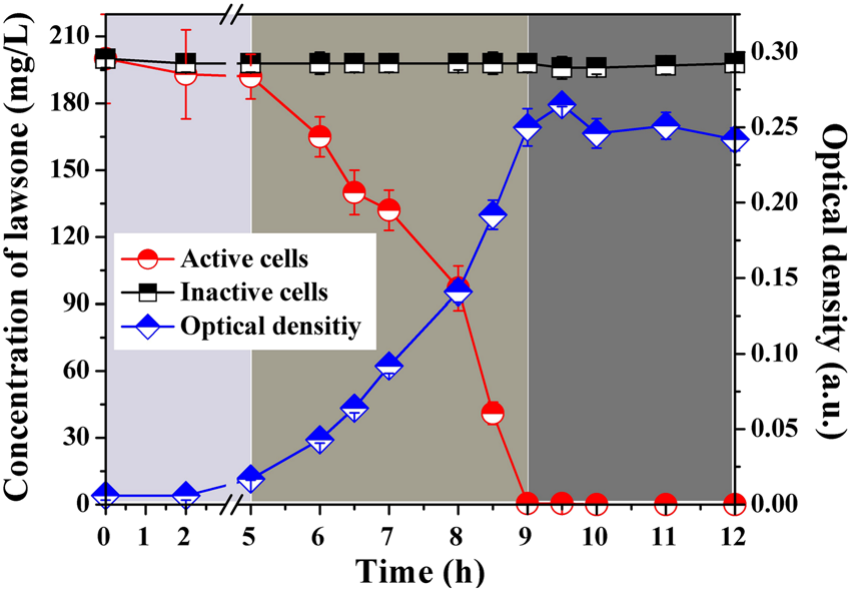
Degradation of lawsone in liquid MSM with the inoculation of active and inactive strain LH-3 and the corresponding growth profile (OD_600_) of active cells. The initial concentration of lawsone was 200 mg L^−1^ and the inoculated amount of LH-3 was 1%, the inactive cells indicated the autoclave-killed cells, the growth curve was divided into three parts as filled with different colors, error bars represented the standard error of three replicates.

As shown in Fig. 3, active LH-3 could completely degrade 200 mg L^−1^ lawsone within 9 h. Correspondingly, the color of the MSM faded away along with the degradation process, indicating the chromophore in lawsone was destructed by strain cells. Meanwhile, there was a great symmetry relationship between the degradation curve and the growth profile. Accordingly, the whole grow period could be divided into three phases: (i) a lag phase (0~5 h), (ii) an exponential phase (5~9 h), and finally (iii) a pseudo-stationary phase (9~12 h)^16, 26^. During the whole process, the OD_600_ increased from 0.02 to 0.25 and the lawsone was simultaneously degraded, indicating the LH-3 could consume lawsone as the sole source of carbon (no other carbon source present in liquid MSM) and energy for its maintenance. Besides, the primary degradation was found during 5~9 h, which corresponded to the exponential phase of LH-3. It was not surprising because the growth and metabolism of strain cells were exuberant in this phase, as confirmed by the growth curve^27^. At the initial of this phase, the concentration of lawsone saturated the bacterial uptake system and the bacteria grow exponentially at their physiologically limited maximum rate. After that, with the increase of the population of bacteria, the lawsone dropped below saturation and the exponential growth ceased^28^.

In addition, no significant change of lawsone concentration was observed in the medium inoculated with inactive LH-3 cells, indicating the negligible adsorption of lawsone by strain cells or flask.

### Effects of environmental conditions on biodegradation

**Figure 4:**
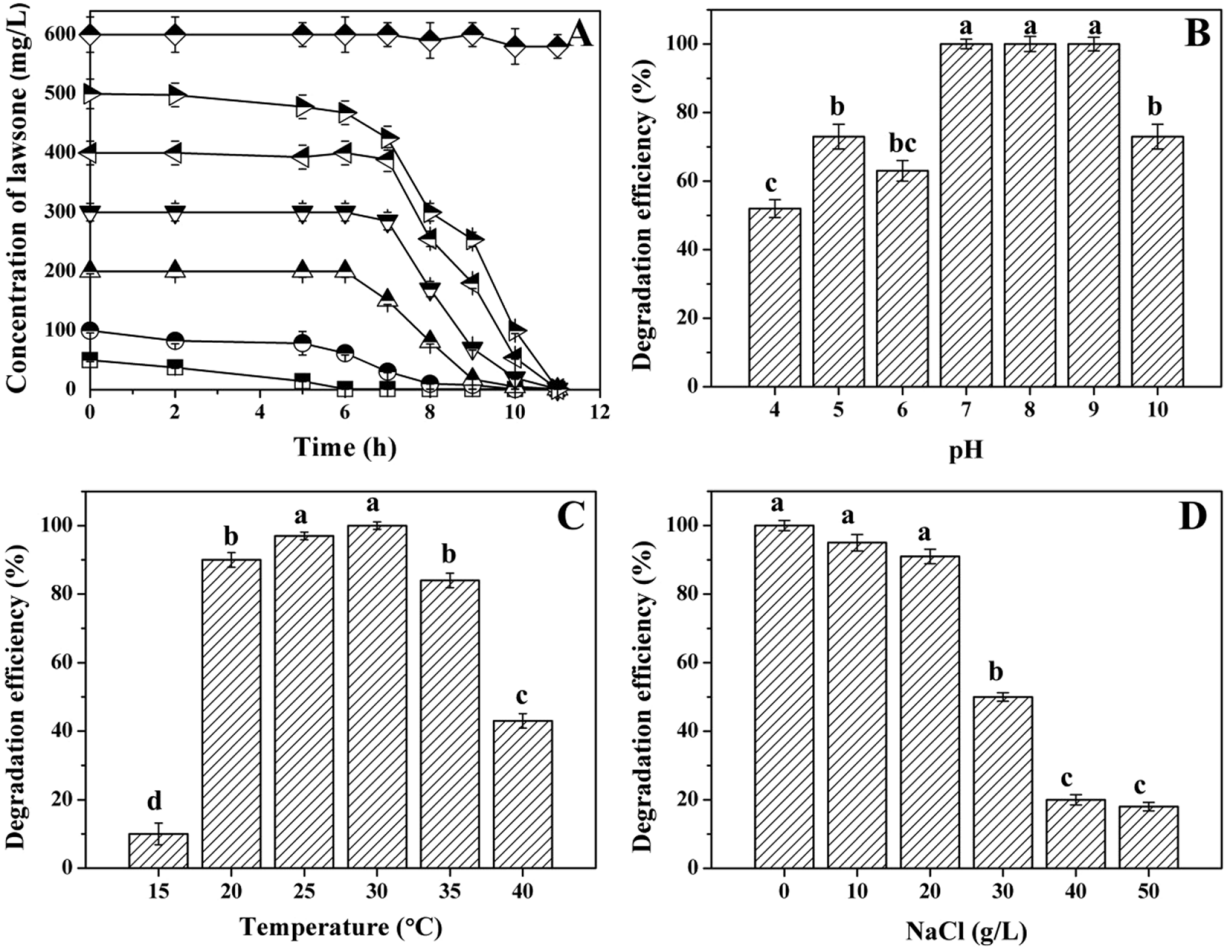
Effects of initial concentration (**A**), pH (**B**), temperature (**C**), and NaCl (**D**) on the biodegradation of lawsone by strain LH-3. The experimental conditions were 200 mg L^−1^ lawsone, 30 °C, pH of 7.0 unless otherwise stated. No NaCl was added in (**A**–**C**). The sampling time was 9 h in (**B**–**D**). The lowercase shown on the top of column indicated the significant analysis conducted by SPSS, *p* < 0.05.

The degradation capability of LH-3 was further evaluated in terms of different concentrations of lawsone. As given in Fig. 4, the degradation could be accomplished within 11 h when the concentration was lower than 500 mg L^−1^, indicating that the strain LH-3 was very effective in degrading lawsone. We noticed that a prolonged lag phase was necessary to achieve the rapid degradation when the initial concentration increased^29^. This was probably due to that the bacterial grew slowly and required an acclimation period before accelerated degradation occurred at high lawsone concentrations^30, 31^. On the other hand, the negative effect (toxicity) of lawsone might occur when the concentration of lawsone was up to 600 mg L^−1^ ^32, 33^. As shown, the concentration was not decreased significantly during 12 h when the concentration of lawsone was 600 mg L^−1^. This is probably due to that the high concentration (over 600 mg/L) of lawsone is toxic to the strain LH-3. In that case, the dilution process might be necessary for the growth of LH-3.

The pH and temperature usually played important roles in the biodegradation process^17^. As shown in Fig. 4B, almost complete degradation of lawsone was achieved when the pH was 7~9. The degradation efficiency was significantly decreased when the pH was lower or higher than this range, indicating the strain cells were sensitive to the pH variations. Moreover, the optimal temperature was found to be 25~30 °C. Extreme conditions (lower than 20 °C and higher than 40 °C) would lead to inactivation of some essential enzymes and proteins which associated with cell growth and the biodegradation^34^. Consequently, the degradation efficiency was significantly inhibited.

Salt at varying concentrations were often present in many types of industrial wastewaters including dye wastewater (as high as 60~100 g/L)^35, 36^. The salt stress could constrain many enzymes of microbial species and reduce cellular activity, resulting in plasmolysis^37^. Consequently, it was very important to evaluate the degradation capability of LH-3 in the presence of salts. As shown in Fig. 4D, the LH-3 could keep its activity until the concentration of NaCl was above 20 g/L, indicating its ability to tolerate salt. Similarly, 90% of reactive blue 160 was decolourized at 20 g/L NaCl within 18 h by using mixed cultures BDN^38^. The results clearly illustrated that LH-3 could degrade lawsone effectively with the presence of salt (0~20 g/L). This attractive character would make it very appropriate in the remediation of lawsone-containing wastewater with high salinity.

### Identification of metabolites

The potential metabolites and the corresponding mass spectrum were shown in Table S1 and Figs S2–4. The prominent protonated molecular ions were found at [M-H]^−^109, [M-H]^−^207, and [M-H]^−^137. The compounds were designated as products A, B, and C, respectively (Table S1 in SI).

The m/z of product A was 109 [M-H]^−^, which had characteristic second-order MS fragment ion peaks at m/z = 81 (−28 Da) and 53 (−56 Da). The fragment ion could be therefore assigned to be CHO and CHOCO. As a result, A was speculated to be catechol (Fig. S2). Product B showed a prominent protonated molecular ion at m/z = 207 [M-H]^−^, and the characteristic second-order MS fragment ion peaks at m/z = 135 (−72 Da), 93 (−114 Da) and 59 (−148 Da) (Fig. S3). Accordingly, the product B was supposed to be 2-hydroxy-4- oxo-chroman-2-carboxylic acid. The product C showed a molecular ion peak at m/z = 137 [M-H]^−^ and characteristic second-order MS fragment ion peaks at m/z = 93 (−44 Da) and 65 (−72 Da) (Fig. S4). Based on these results, product C was recognized as salicylic acid. To verify these speculations, the available authentic standard compounds (catechol and salicylic acid) were analyzed by HPLC and the retention times were in consistent with those of product A and C, respectively. Similarly, Wessendorf *et al*.^22^ also detected products A and C during the biodegradation process of lawsone by *Pseudomonas putida* L2. Our results agreed well with the previously reported results. However, the product B was reported for the first time for the biodegradation of lawsone. This finding might be valuable to be a complement to the degradation routine of lawsone. Unfortunately, the product B was not further confirmed by the authentic standard compound because it was not commercially available.

**Figure 5:**
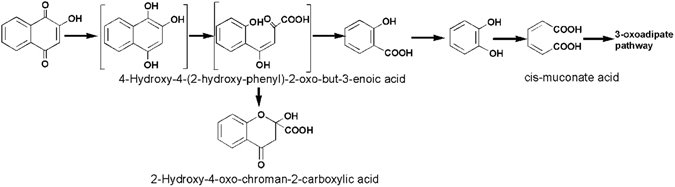
Proposed metabolic pathway of lawsone by strain LH-3. (The compounds shown in the brackets were not detected but speculated to be present in the system).

According to the identified metabolites, a tentative metabolic pathway was proposed. As shown in Fig. 5, the lawsone was initially reduced to 1,2,4-trihydroxynaphthalene. Noting that, the hydroxyl could increase the electron cloud density of naphthalene ring. As a result, the 1,2,4-trihydroxynaphthalene was relatively unstable and not detected in this study. Then, the fission of the naphthalene ring occurred, leading to the generation of 4-hydroxy-4-(2-hydroxy-phenyl)-2-oxo-but-3-enoic acid. The 4-hydroxy-4-(2-hydroxy-phenyl)-2-oxo-but-3-enoic acid was immediately converted into 2-hydroxy-4-oxo-chroman-2-carboxylic acid through the keto-enol tautomerism reaction^39^. Subsequently, salicylic acid was produced by the removal of side chain. After that, salicylic acid was further degraded to catechol, which was probably cleaved in the ortho-position by catechol 1,2-dioxygenase^40^. Finally, the resultant cis-muconate acid was mineralized through the 3-oxoadipate pathway^41^.

### Degradation of lawsone by immobilized LH-3

Immobilized cell technology could enhance the efficiency and effectiveness of specific functional strains^42, 43^. The loss of freely suspended cells during the application could be well prohibited by immobilization technique. In this study, alginate gel was used as the immobilization matrix due to its low toxicity and gelled easily^44, 45^. However, it suffered from the poor mechanical strength and mass transfer^46^. We supposed that the introduced biochar could enhance the mechanic strength and specific surface area of the final gel beads, which were favorable for the mass transfer.

**Figure 6:**
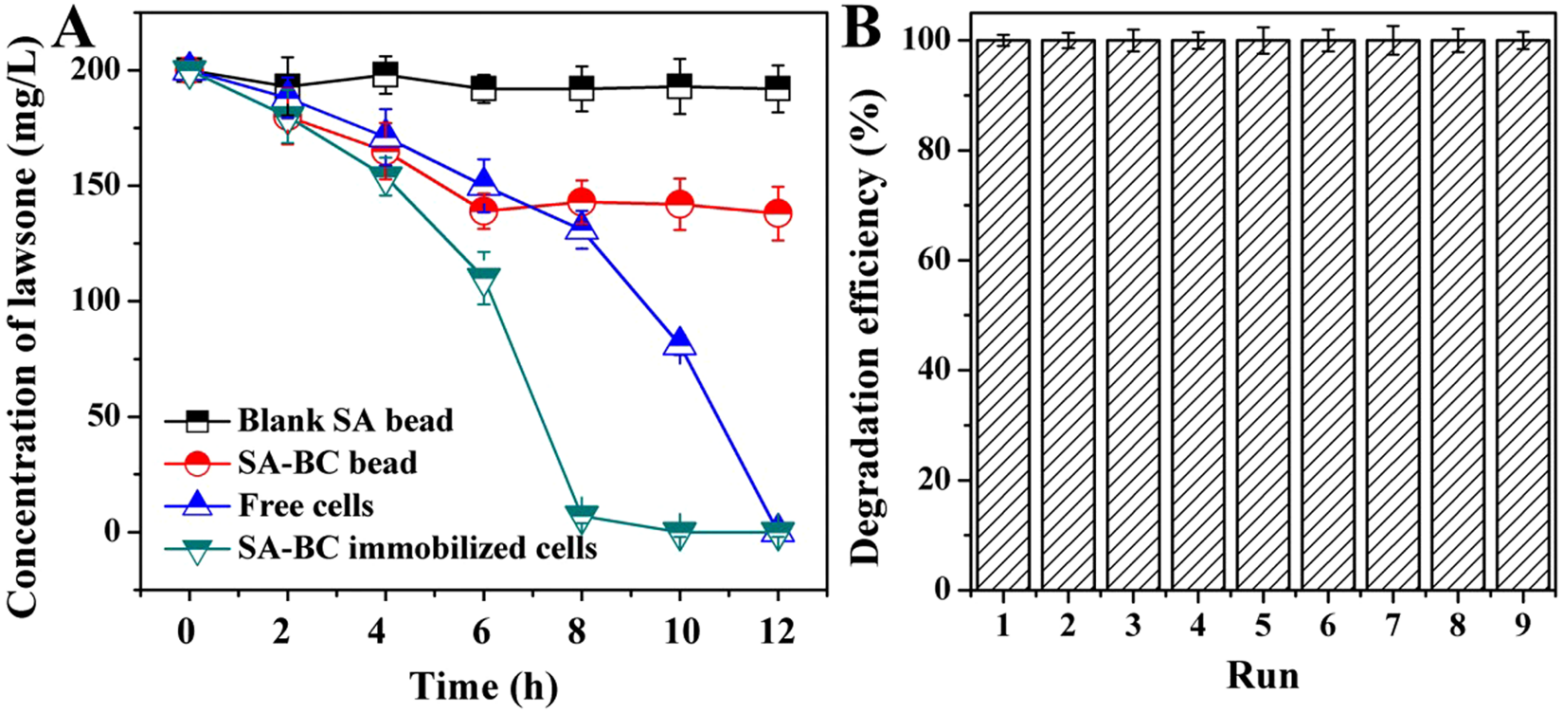
(**A**) Variation of the concentration of lawsone in the presence of blank alginate bead (■), alginate-biochar bead (), free LH-3 cells (), and alginate-biochar immobilized cells (); (**B**) Degradation efficiencies of alginate-biochar immobilized LH-3 cells towards lawsone in 9 successive runs. (The experimental conditions were given in the Section 2.3 and were 200 mg L^−1^ lawsone, 30 °C, pH of 7.0 unless otherwise stated. The inoculation quantities of free and immobilized cells were both 2% (v/v). The duration of each run in B was 12 h).

As shown in Fig. S5, the alginate beads immobilized with LH-3 cells were black in color due to the introduced biochar. The SEM image clearly illustrated that the beads possessed well-developed porous structure. LH-3 cells were uniformly dispersed on the inter-walls of the spherical beads. As expected, under the same inoculation quantity of strain cells, immobilized LH-3 performed the best degradation capacity (Fig. 6A), which was in well agreement with previous studies^46^. The enhanced degradation capacity might be attributed to two reasons. Firstly, the biochar was known for the high affinity towards organic pollutants according to its high specific surface area and porous structure^47^. Accordingly, it could be suggested that the biochar enhanced the removal of lawsone through adsorption mechanism. This deduction could be confirmed by the distinct difference between the lawsone concentration curves of blank alginate bead and the alginate-biochar bead in Fig. 6A. Secondly, the well-structured spherical bead provided a micro-zone for strain cells. The immobilized cells could be protected from the environmental shocks such as hydraulic shock. Moreover, the good biocompatibility of biochar even promoted the colonization and growth of bacteria in the spherical beads^48^. As a result, the population and the activity of the immobilized cells were higher than the freely suspended cells^49^. In addition, the stability of the immobilized cells in successive degradation runs was evaluated. It was clearly shown in Fig. 6B that the immobilized cells performed an excellent stability during the investigated 9 runs. The spherical beads could keep their structure without visible collapse. The outstanding stability would make it more economically attractive in the industrial-scale application.

### Degradation of lawsone-containing artificial wastewater in SBR

**Figure 7:**
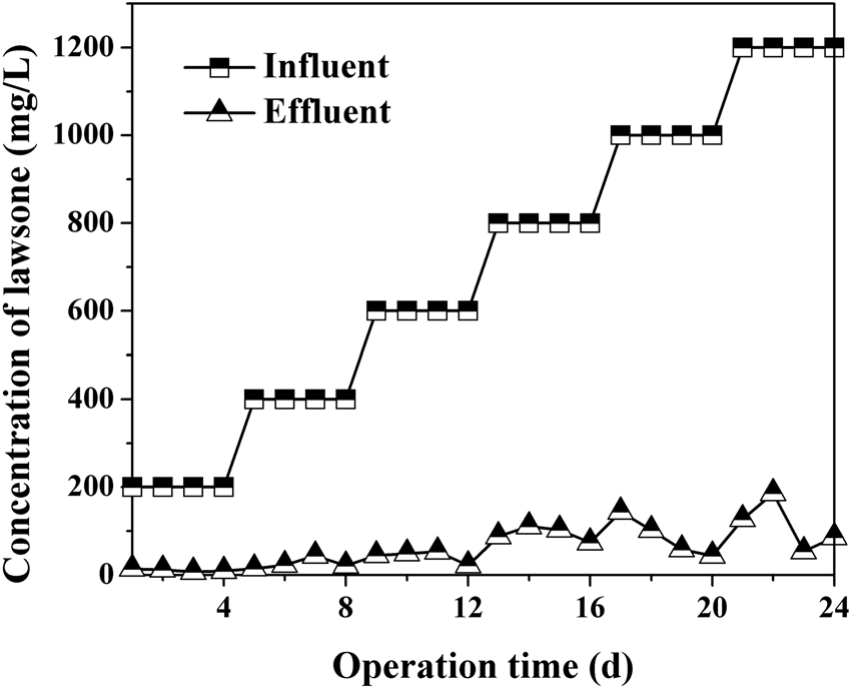
Degradation profiles of lawsone in artificial wastewater by laboratory scale SBR.

As shown in Fig. 7, the SBR could effectively remove lawsone from the artificial wastewater in a relatively long period. In addition, the reactor possessed an excellent resistance to impact load. The removal efficiencies were almost over 90%. The positive results might indicate that the immobilized LH-3 cells successfully survived in the SBR. This finding was very important because the failure of a bioaugmentation system was usually attributed to the competition with indigenous bacteria^50^. To verify this speculation, the investigation on microbial community dynamics would provide more helpful information.

**Figure 8:**
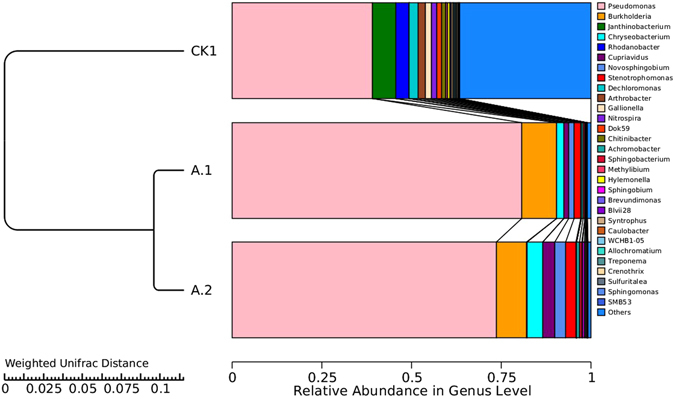
Relative abundance in genus level among the different samples.

After cleaning the raw reads, a total of 37530, 30038, and 42758 effective sequence tags in the samples of CK1, A1, and A2 were obtained, respectively. The obtained effective tags were classified to operational taxonomic units (OUT) by the software UCLUST and finally classified by the Ribosomal Database Project classifier (RDP). The results were given in Table 1 and Fig. 8, respectively.

**Table 1:** Summary of the alpha diversity estimates in the samples.

Sample	Sequence number	OTU number	Shannon	Chao l	Observed_species	PD_whole_tree
CK1	37530	667	5.186478	671.4416	658	54.82298
A1	30038	150	1.286411	160.1364	121	13.86755
A2	42758	113	1.704558	89.04762	80	8.8706

The alpha diversity indexes listed in Table 1 clearly showed the variation of the microbial community in the different samples. Among them, shannon index accounted for both richness and evenness of OTUs. Chao 1 index was used to estimate the number of OTUs, indicating the richness of the OTUs. The phylogenetic diversity (PD) whole tree was a diversity index calculated based on the phylogenetic tree. Table 1 indicated that the Shannon index value was greatly decreased in A1 and A2 samples when comparing with the CK1 sample. Similarly, the Chao 1 and PD whole tree index were also significantly reduced. All the results clearly revealed that the biodiversity of the sludge decreased along with the operation of SBR. The possible reason was that the introduction of the high concentration of the pollutant lawsone inhibited the growth of some microorganisms. Some heterotrophic bacteria might also be greatly suppressed due to the lack of available carbon source.

Figure 8 further revealed the abundance of microorganisms in genus level. The *Pseudomonas* (38.89%), *Janthinobacterium* (6.94%), and *Rhodanobacter* (4.17%) occupied quite a large proportion among all the genus in the sample CK1. The abundance of *Pseudomonas* was greatly enhanced in A1 (80.56%) and A2 (73.61%), probably indicating that the LH-3 cells adapted to the aquatic environment and became the dominant strain in the system. However, the latter genus disappeared. Besides, the *Burkholderia* (9.72% in A1 and 8.33% in A2), *Chryseobacterium* (2.44% in A1 and 4.17% in A2) and other genus also played more important roles along with the operation of SBR. *Burkholderia* sp. had been demonstrated to be able to degrade many kinds of organic pollutants including ethylene diamine tetra acetic acid^51^, pyrene^52^, polychlorinated biphenyl^53^, and etc. Recently, the degradation of organochlorine pesticides by *Chryseobacterium* sp. PYR2 was also reported^54^. The experimental results indicated that the *Burkholderia* sp. and *Chryseobacterium* sp. bacteria could survive in the aquatic system with high concentration of lawsone.

## Materials and Methods

### Materials and media

The activated sludge used for bacterial isolation was collected from a pesticide factory located in Jiangsu, China and was concentrated by natural settling. Lawsone (98%) was purchased from Aladdin (Shanghai, China). Chromatographic grade methanol was purchased from Sigma-Aldrich (St. Louis, USA). Molecular biological reagents were obtained from Takara Biotechnology Co. Ltd. (Dalian, China).

Luria-Bertani (LB) and mineral salts medium (MSM) were prepared according to our previous studies^16, 17^. Specifically, the LB was consisted of 10.0 g NaCl, 10.0 g peptone and 5.0 g yeast extract per liter water. MSM was consisted of 1.0 g (NH_4_)_2_SO_4_, 1.5 g K_2_HPO_4_, 0.5 g KH_2_PO_4_, 0.2 g MgSO_4_·7H_2_O, and 1.0 g NaCl per liter water. The pH of LB and MSM were adjusted to be 7.0. Except the liquid media, 1.8% agar powder was added to the above mentioned media to make the solid media. The prepared media were autoclaved at 121 °C for 20 min prior to use. Lawsone stock solution (1000 mg/L in water) was added into the above media if necessary.

### Isolation and identification of the lawsone-degrading bacteria

Typically, 5 mL concentrated activated sludge was added into 100 mL liquid MSM containing 100 mg L^−1^ lawsone in 250 mL flask and incubated at 30 °C (160 rpm). Several days later, the liquid medium became visually turbid and the color (orange of lawsone) faded away. Then, 5% (v/v) of the above liquid was transferred into the same volume MSM containing 200 mg L^−1^ lawsone and incubated as above mentioned. The whole transfer process was repeated for 4 times successively with the concentration of lawsone in MSM gradually increased to 500 mg L^−1^. Finally, pure strain was obtained on the solid agar medium containing 100 mg L^−1^ lawsone by using streak-plate method. Over 10 colonies were picked out as the candidates and their degradation capacities towards lawsone were compared. Among them, we selected one strain possessing the highest degradation capacity and designated it as LH-3.

The identification of strain LH-3 was done according to the Bergey’s Manual of Determinative Bacteriology^55^. The 16S rRNA gene was amplified by PCR using 27F and 1492R as primer set^56^. The PCR products were analyzed for sequencing analysis at Nanjing Jinsirui Biological Technique Co. (Nanjing, China). This sequence was then used as query for BLAST homology searches which was performed using Clustal X 2.1 and Mega 5.05. A phylogenetic tree was constructed by the Neighbor-Joining method and the data set was bootstrapped 1000 times^16^. The GenBank accession number for 16S rRNA sequence of strain LH-3 was KX225456. The cell morphology of the isolate was observed by transmission electron microscopy (TEM, HITACHI H-7650, Japan).

### Biodegradation of lawsone by freely suspended LH-3 cells

The newly isolated strain was pre-cultured until late logarithmic growth phase in LB medium prior to the biodegradation experiments. Then the strain cells were harvested (centrifuged at 8000 rpm for 1 min), washed (twice with sterilized water), and re-suspended in liquid MSM to make the optical density (at 600 nm, OD_600_) of ~1.0. The obtained suspension was designated as LH-3 seed liquid.

Four milli-liter seed liquid was injected into 400 mL liquid MSM (1% inoculum, v/v) supplemented with 200 mg L^−1^ lawsone to initiate the biodegradation process. At regular time intervals, samples were collected to detect the biomass and residual lawsone. Noticeably, the autoclave-killed cells were used as negative controls^57^.

To gain more details on the biodegradation behavior, the effects of initial pollutant concentration, pH value, temperature, and NaCl were investigated, respectively. The significance analysis among the experimental results was conducted with SPSS software.

### Preparation and stability test of immobilized LH-3 cells

LH-3 strain cells were immobilized in alginate gel bead with biochar as additive in this study. The biochar derived from masson pine tree was prepared according to a previous study^48^. During the preparation process, the biochar was mixed with the cell suspension prior to the gelation. The gelation procedure was performed according to our previous study^49^. The inoculum of LH-3 cells during the immobilization process was 2% and the dose of biochar was 2.5 g/L. The diameter of the resultant alginate bead was 4.0 ± 0.2 mm. To check the immobilized cells in the alginate bead, the scanning electron microscopy (SEM) observation was conducted.

For the stability test, the spherical beads were filtered from the liquid MSM containing lawsone after each cycle (12 h) and washed gently with sterilized water prior to the next run.

### Degradation of lawsone by immobilized LH-3 cells in sequencing batch reactor

To evaluate the application of immobilized cells in treating lawsone containing wastewater, a laboratory scale cylindrical sequencing batch reactor (SBR) was developed with plexiglass. The diameter and the height of the reactor were 10 cm and 40 cm, respectively. Noticeably, the working volume of the SBR was 3 L. The artificial wastewater consisted of 1.0 g/L (NH_4_)_2_SO_4_, 1.5 g/L K_2_HPO_4_·3H_2_O, 0.5 g/L KH_2_PO_4_, 0.2 g/L MgSO_4_·7H_2_O, and 200 mg/L lawsone (increased to 1200 mg/L step by step) was pumped into the SBR by a peristaltic pump (flow rate: 600 mL/min). 150 mL activated sludge (dry weight: 1.58 g) combined with a certain amount of immobilized LH-3 cells (inoculum: 0.4%) were then inoculated into the SBR all at once to initiate the treatment process. A typical operation cycle (24 h) of the SBR was as follows: 5 min for inflow, 23.5 h for aeration (air flow: 3 L/min), 12 min for settling, 3 min for decant, and 10 min for idling. Noting that, the effluent was withdrawn through the outlet ports located at the medium height of the column reactor, resulting in volumetric exchange ratio of 50%. The SBR was operated in open condition at room temperature (20 °C) for 24 cycles (24 d). The concentrations of lawson in influent and effluent were recorded after each cycle. To investigate the dynamic of the microbial community, the raw activated sludge and the liquid mixtures in SBR after the 16^th^ and the final (24^th^) cycles were collected and designated as CK1, A1, and A2, respectively. The genomic DNA of the samples was extracted with the TIANamp Bacteria DNA Kit (Tiangen Biotech Co., Ltd., Beijing). The extracted DNA was sent to TGS, Shenzhen for the high-throughput sequencing (Illumina HiSeq PE250).

### Identification of Metabolites

One milli-liter LH-3 seed liquid was injected into 100 mL liquid MSM containing 500 mg L^−1^ lawsone and incubated at 30 °C. Aqueous samples were collected at 1 h intervals and subjected for the LC-MS analysis. The metabolites were confirmed with authentic standard compounds with respect to both retention time and MS spectra.

### Analytical Methods

The OD_600_ was used to evaluate the amount of LH-3 cells in suspension and measured by a PHILES D-8 spectrophotometer (China). The concentration of lawsone was analyzed by HPLC (LC-20A, SHIMADZU, Japan) equipped with a reverse-phase C18 column (250 mm × 4.6 mm, 2 µm) and a UV detector (detection wavelength: 269 nm). The mobile phase was methanol/water/formic acid (70: 30: 0.1, v/v/v), and the flow rate was 1.0 mL min^−1^. External standard method was used to determine the final concentration.

The identification of metabolites was conducted with a liquid chromatograph-triple quadrupole mass spectrometer (LC-QqQ-MS, Agilent G6410B). The mass spectrometer was operated in negative electrospray ionization (ESI) mode. The ESI conditions were as follows: a gas temperature of 350 °C, a capillary voltage of 4.0 kV, a nebulisation pressure of 30.0 psi, and a gas flow rate of 10.0 L min^−1^. MS/MS conditions were as follows: a fragmentor voltage of 90 V and collision energy of 10–25 eV.

## Conclusions

In summary, an effective lawsone-degrading strain (LH-3) was isolated from activated sludge and characterized. The sequence results of 16S rRNA revealed that the strain was belong to the *Pseudomonas taiwanensis*. The strain LH-3 could achieve complete degradation in 11 h with the inoculation of 1% when the concentration of lawsone was lower than 500 mg/L. The optimal pH and temperature were determined as 7~9 and 25~30 °C, respectively. More importantly, the LH-3 could keep its high efficiency under high salt concentration (as high as 20 g/L), making it very attractive in remediation of real wastewater with high salinity. A possible degradation pathway was tentatively proposed through LC/MS anslysis. Finally, the LH-3 strain cells were immobilized with alginate gel and biochar as additive and showed excellent stability in degrading lawsone. LH-3 could adapt to the complex environment and become to the dominant bacteria along with the operation of SBR. Overall, LH-3 could be a competitive candidate for the remediation of lawsone-containing industrial wastewater.

## Electronic supplementary material

Supplementary Information
