# Supplementary material for: Biodegradation of 2-hydroxyl-1,4 naphthoquinone (lawsone) by Pseudomonas taiwanensis LH-3 isolated from activated sludge
Source: Sci Rep. 2017 Jul 28;7:6795. doi: 10.1038/s41598-017-06338-1 (PMC5533781; doi:10.1038/s41598-017-06338-1)
Supplement: Supplementary file 1 — Supplementary Information [file 41598_2017_6338_MOESM1_ESM.pdf]

Supporting information

Biodegradation of 2 - hydroxyl - 1,4 naphthoquinone (lawsone) by *Pseudomonas taiwanensis* LH-3 isolated from activated sludge

Li Yang[a,b], Tianming Cai[a], Dahu Ding[a], Tianjin Cai [a], Canlan Jiang[a], Hua Li[a], Qian Yang[a], Liwei Chen[a,*]

a College of Resources and Environmental Sciences, Nanjing Agricultural University, Nanjing 210095, China

b College of Geographic and Biologic Information Nanjing University of Posts and Telecommunications , Nanjing, ,210023, China

***Corresponding author: Liwei Chen**

Postal address: a 1 Weigang, Xuanwu District, Nanjing, 210095, People’s Republic of China

b 9 Yuen Road in Qixia District of Nanjing City, Jiangsu Province, China

E-mail address: clw@njau.edu.cn

Fax: +86-25-84395002

Tel: +86-25-84395002

Table 1 Summary of the degradation intermediates detected in this study.

No.	Retention time(min)	m/z	MS/MS	Proposed compound
1	6.416	109	81, 53	catechol
2	9.963	207	135, 93, 59	2-Hydroxy-4-oxo-chroman-2-carboxylic acid
3	11.022	137	93, 65	salicylic acid

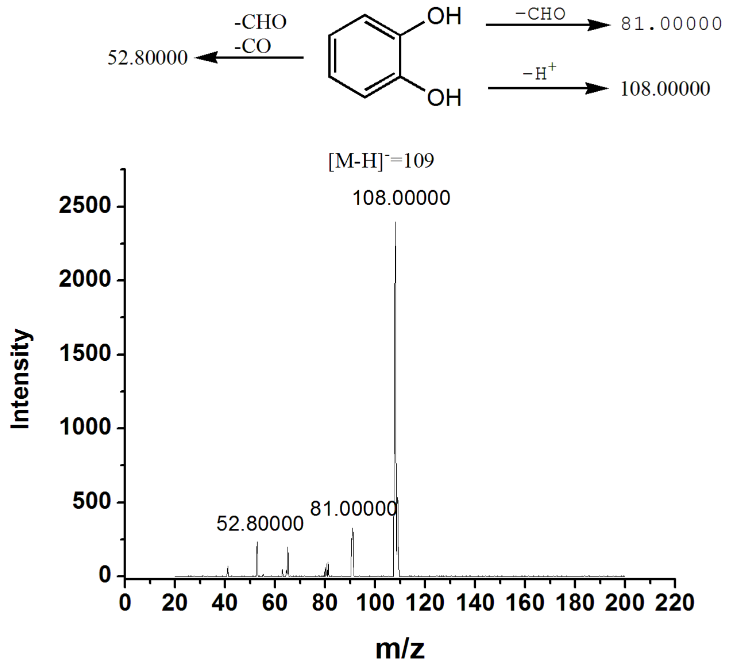

Fig. S1 Second-order MS fragment of product A.

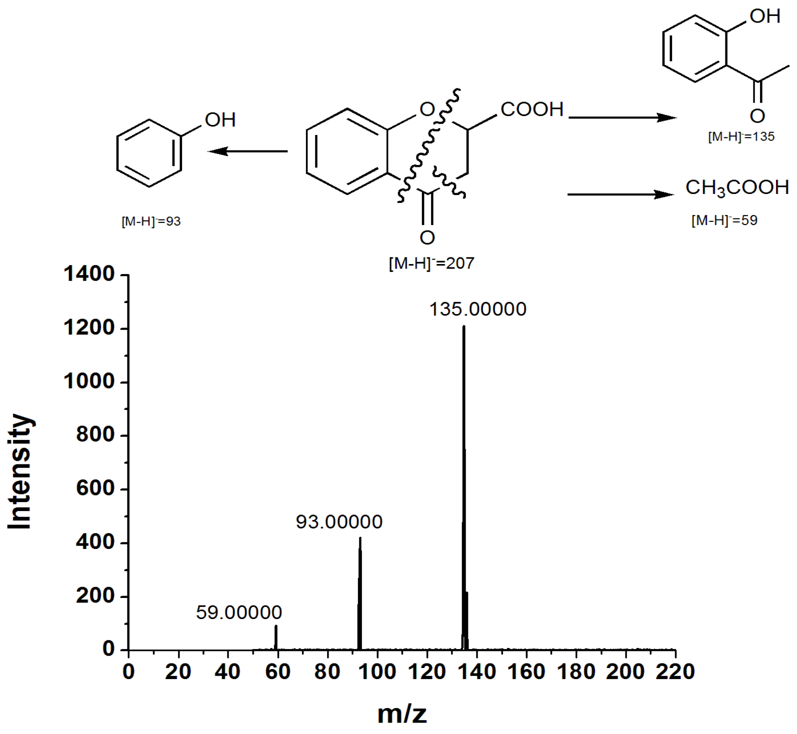

Fig. S2 Second-order MS fragment of product B.

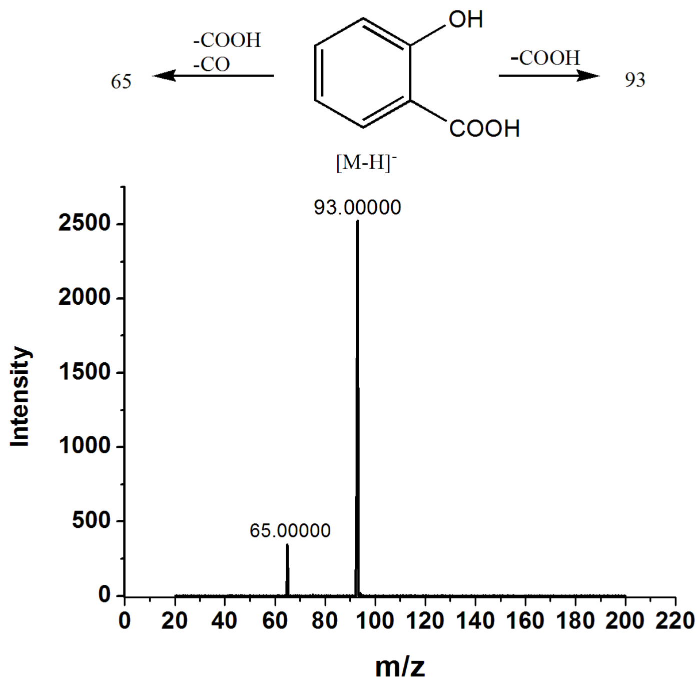

Fig. S3 Second-order MS fragment of product C.

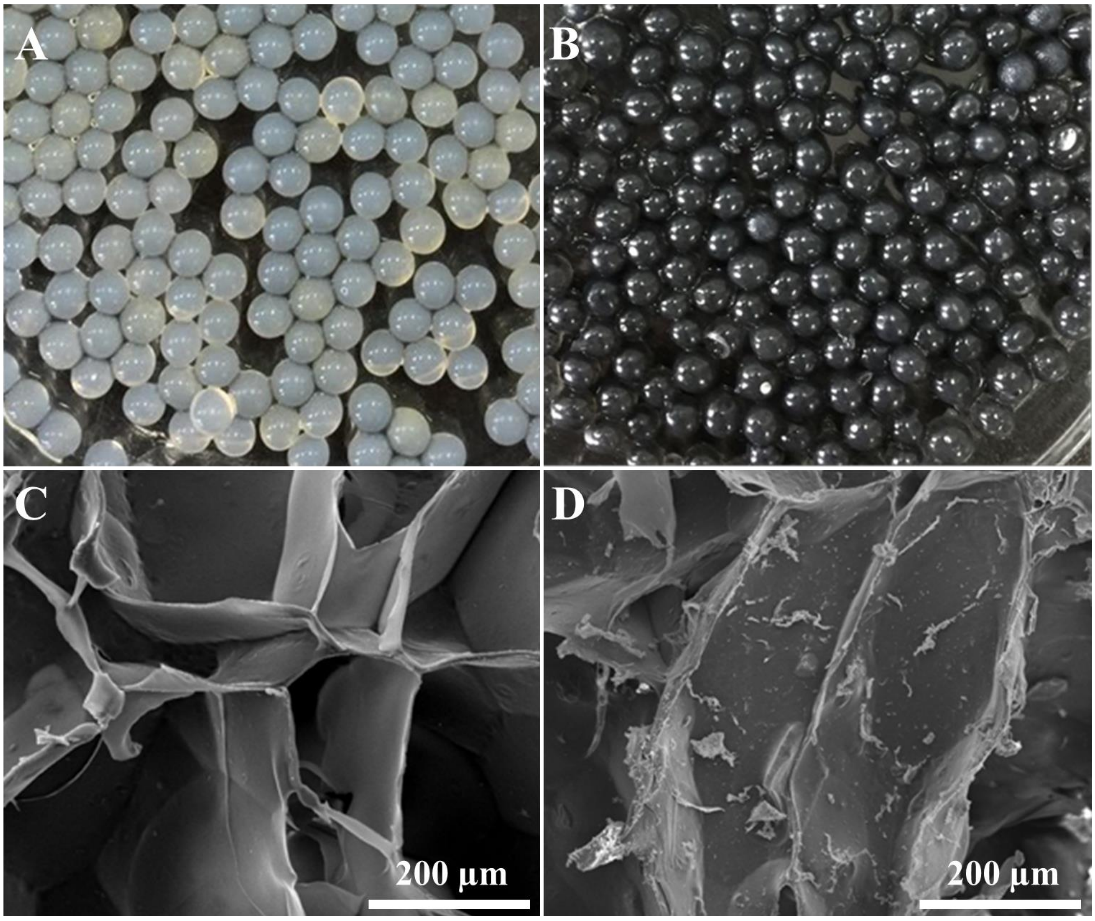

Fig. S4 Photographs and SEM images of the blank alginate beads (A and C) and the biochar

enhanced alginate beads immobilized with LH-3 cells (B and D).
